# Adaptation of a Differential Scanning Calorimeter for Simultaneous Electromagnetic Measurements

**DOI:** 10.3390/s24186077

**Published:** 2024-09-20

**Authors:** John W. Wilson, Mohsen A. Jolfaei, Adam D. Fletcher, Carl Slater, Claire Davis, Anthony J. Peyton

**Affiliations:** 1 Department of Electrical and Electronic Engineering, University of Manchester, Manchester M13 9PL, UK; adam.fletcher@manchester.ac.uk (A.D.F.); a.peyton@manchester.ac.uk (A.J.P.); 2 Warwick Manufacturing Group, University of Warwick, Coventry CV4 7AL, UK; mohsen.jolfaei@warwick.ac.uk (M.A.J.); c.d.slater@warwick.ac.uk (C.S.); claire.davis@warwick.ac.uk (C.D.)

**Keywords:** electromagnetic, nickel, DSC, high temperature

## Abstract

Although much information can be gained about thermally induced microstructural changes in metals through the measurement of their thermophysical properties using a differential scanning calorimeter (DSC), due to competing influences on the signal, not all microstructural changes can be fully characterised this way. For example, accurate characterisation of recrystallisation, tempering, and changes in retained delta ferrite in alloyed steels becomes complex due to additional signal changes due to the Curie point, oxidation, and the rate (and therefore the magnitude) of transformation. However, these types of microstructural changes have been shown to invoke strong magnetic and electromagnetic (EM) responses; therefore, simultaneous EM measurements can provide additional complementary data which can help to emphasise or deconvolute these complex signals and develop a more complete understanding of certain metallurgical phenomena. This paper discusses how a DSC machine has been modified to incorporate an EM sensor consisting of two copper coils printed onto either side of a ceramic substrate, with one coil acting as a transmitter and the other as a receiver. The coil is interfaced with a custom-built data acquisition system, which provides current to the transmit coil, records signals from the receive coil, and is controlled by a graphical user interface which allows the user to select multiple excitation frequencies. The equipment has a useable frequency range of approximately 1–100 kHz and outputs phase and magnitude readings at a rate of approximately 50 samples per second. Simultaneous DSC-EM measurements were performed on a nickel sample up to a temperature of 600 °C, with the reversable ferromagnetic to paramagnetic transition in the nickel sample invoking a clear EM response. The results show that the combined DSC-EM apparatus has the potential to provide a powerful tool for the analysis of thermally induced microstructural changes in metals, feeding into research on steel production, development of magnetic and conductive materials, and many more areas.

## 1. Introduction

Electromagnetic measurements can provide a rich source of information on heat-induced microstructural changes in metals, from small-scale laboratory measurements studying phenomena such as recovery and recrystallisation [1] and ferrite grain size [2] in specific steels to permanent industrial installations monitoring processes such as the production of strip steel [3,4,5]. However, EM measurements in isolation cannot fully characterise the complex microstructural interactions involved. EM measurements are frequently supplemented by optical metallography and/or scanning electron microscopy (SEM) with electron backscattered diffraction (EBSD) scans to visualise and quantify the material microstructure [6]. Although it is possible to generate EBSD images at high temperatures [7], these techniques are generally only used at room temperature before and after heating to characterise the change in microstructure, which can then be related to the dynamic changes in the EM signal.

A DSC uses a different approach to characterise heat-induced microstructural changes. Differential scanning calorimetry is used to measure the heat flow associated with physical and chemical changes in a sample under varying thermal conditions. The method monitors the heat flux into or out of the sample, which can be recorded through various techniques, including the temperature difference between the sample and a reference material, as well as other methods like power compensation or Peltier-based sensors [8]. This enables the detection of endothermic and exothermic transitions such as melting, crystallisation, and decomposition, making DSC invaluable for analysing material properties such as purity, stability, and composition.

DSC measurements are frequently augmented by data from other techniques to allow full interpretation of microstructural changes. Imaging techniques such as optical microscopy [9] and confocal scanning laser microscopy [10] are commonly used, and techniques like thermogravimetric analysis [11] or dilatometry [12] can provide a more comprehensive understanding of material properties by correlating thermal changes with mass loss or dimensional changes, respectively [13]. Novel techniques have also been trialled, such as direct measurement of the electrocaloric effect in ferroelectric ceramics using a spring-loaded high voltage electrode applied to the sample in the DSC during the heating cycle [14]. The relationship between polarization and electric field, known as the P(E) hysteresis loop, was also calculated from these measurements at temperatures up to 180 °C.

When applied to steels, DSCs have been used to study a wide range of phenomena, including melting and solidification behaviour [10,15], phase transformations [16,17,18], Cu precipitation [19], precipitation hardening [20], and intragranular ferrite transformations [21,22]. More complex steels, such as alloy or stainless steels containing significant amounts of alloying elements (chromium, nickel, etc.), exhibit thermal events that involve significant heat release or absorption which are clearly detectable by DSC [23]. However, the use of DSC heat flow measurements to study steel, particularly simple carbon steel, does have some limitations. There are typically several transformations and microstructural changes that occur over a range of temperatures, which can make detection in the DSC challenging [23], but some of these changes will invoke a strong EM response [24]; therefore, EM measurements may provide a better indication.

An alternate approach is to incorporate EM measurement apparatus into a DSC to provide simultaneous complementary information during heating and cooling. This paper discusses how a Q2000 DSC from TA Instruments [25] has been modified to include a ceramic coil for simultaneous non-contact electromagnetic measurements. This instrument is typical of DSC equipment, so it expected that the techniques presented here could be adapted and applied to many different DSC machines. The electromagnetic measurement system was first tested at room temperature using various samples produced from metals with differing magnetic permeability and electrical conductivity values. A sensor coil assembly was then built into the DSC for simultaneous electromagnetic and heat flow measurements, with the results plotted against temperature for comparison.

## 2. Materials and Methods

### 2.1. Development of Electromagnetic Measurement System and Modification of DSC

**Figure 1:**
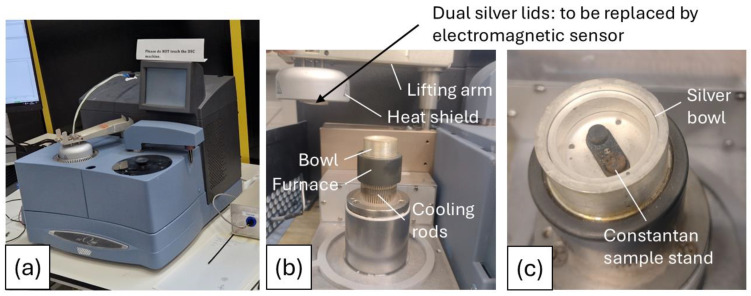
(**a**) Q2000 DSC, (**b**) furnace and bowl, (**c**) close-up of bowl.

Figure 1a shows the device chosen for installation of the electromagnetic sensor. Unlike many DSCs, the furnace for this system is positioned underneath the sample, allowing full access to the sample under inspection from above. As shown in Figure 1b, the bowl containing the sample is sealed by dual silver lids positioned automatically by a lifting arm with extra thermal insulation provided by a dome-shaped heat shield. This design presents the opportunity to replace the dual silver lids with a ceramic electromagnetic sensor assembly, allowing the DSC to function normally using the automated lifting arm while simultaneously gathering electromagnetic data. Figure 1c shows a close-up view of the DSC bowl, indicating the silver bowl and constantan sample stand/sensor housing where the samples are positioned during testing.

**Figure 2:**
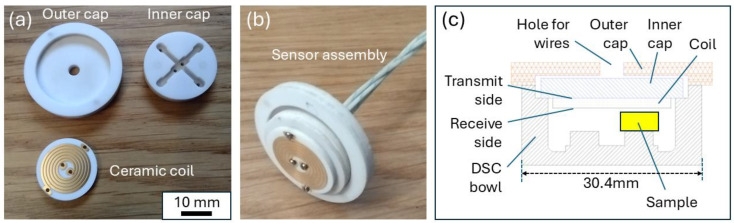
(**a**) Ceramic parts for sensor assembly, (**b**) complete sensor assembly, (**c**) cross-sectional drawing of DSC coil installation.

Figure 2a shows the EM sensor coil designed for the DSC installation. Printed circuit board fabrication techniques were used to print copper coils onto a 1.5 mm thick, 20 mm diameter alumina disc. A 5.75 turn coil was printed on each side of the disc in 140 µm thick copper with ENEPIG (Electroless Nickel Electroless Palladium Immersion Gold) surface plating, resulting in a finished disc approximately 1.7 mm thick. Two more ceramic discs were designed and fabricated to produce the sensor assembly shown in Figure 2b. The coils are connected to the inner cap using M1 titanium machine screws, which also provide an electrical connection between the coils and four sections of 0.5 mm diameter fibreglass-insulated chromel wire taken from a type K thermocouple extension cable.

Figure 2c shows a cross-sectional drawing of the sensor assembly installed in the DSC bowl. The coil is suspended below the inner cap, with the coil on the upper side of the alumina disc acting as the transmitter and the coil on the lower side of the disc, closest to the sample, acting as the receiver. It was decided that a 3 mm sample height would be used for all the tests, resulting in a 0.88 mm gap (lift-off) between the coil and the surface of the sample. Figure 1c shows the DSC bowl for the installation. The bowl itself is predominantly made from silver, the sample stand from constantan, and some chromel [25].

Although the minimal lift-off between the receive coil and the sample surface should maximise the signal measured from the sample and minimise the signal from the metal in the DSC bowl, there are some potential drawbacks to this arrangement. Firstly, the temperature of the sample is measured on the underside of the sample using a thermocouple mounted in the sample stand, whereas the electromagnetic measurements are from the top of the sample. This could lead to some discrepancy between the measured temperature and the temperature of the part of the sample where the electromagnetic measurements take place. Secondly there is a 0.9 mm gap around the whole circumference of the inner cap and the bowl, leading to some difficulty ensuring repeatable positioning of the coil with respect to the sample. Lastly, the lift-off is entirely determined by the height of the sample, so any variation in sample height will also vary lift-off and degrade measurement accuracy.

**Figure 3:**
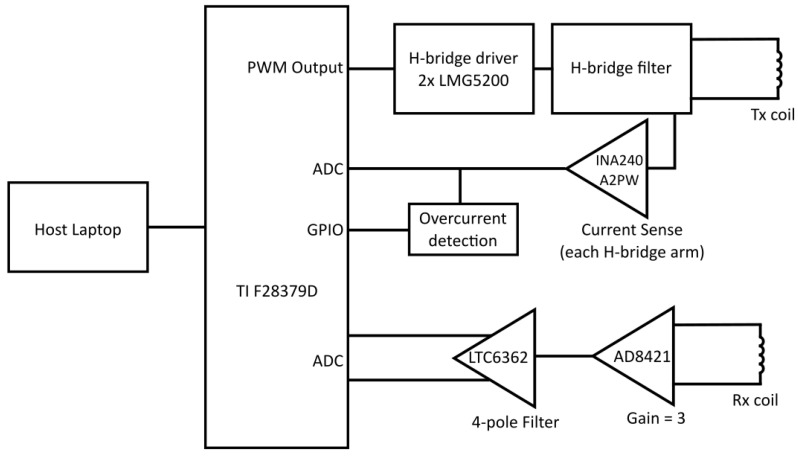
Block diagram of the EM measurement hardware.

The EM sensing coils are controlled by data acquisition hardware and software developed at the University of Manchester. The equipment has a useable frequency range of approximately 1–100 kHz and outputs phase and magnitude readings at a rate of approximately 50 samples per second. The EM hardware is based around a C2000 real-time microcontroller from Texas Instruments, specifically the TMS320F28379D [26]. This is a 32-bit digital signal processor comprising two C28x CPUs, two control law accelerator (CLA) modules, configurable 12- or 16-bit ADCs, and 12-bit DAC and PWM outputs. A block diagram of the signal conditioning chain is shown in Figure 3.

**Figure 4:**
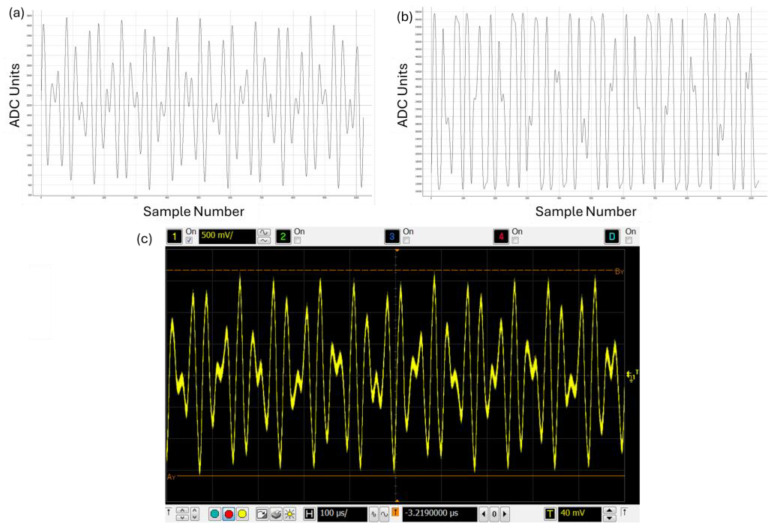
A standard transmit waveform comprising 29th and 41st system harmonics. (**a**) Waveform generated by the system. (**b**) Transmit current measured by the system current sense ADC. (**c**) Voltage waveform output to the coil recorded on an external oscilloscope.

The transmit signal is configurable and can consist of up to 15 component harmonics with individually settable relative gains and phases. A PWM waveform is generated from the transmit signal by the DSP and amplified by two half-bridge driver modules (LMG5200). This is converted to an analogue waveform through an RC filter network and output to the transmit coil. One of the primary waveforms used in this work is shown in Figure 4 and detailed in Table 1, with the component harmonics given as multiples of the system base frequency (976 Hz). A INA240A2PW amplifier on each arm of the H-bridge output provides current sensing, which allows the transimpedance to be calculated and also provides overcurrent and short circuit protection. The receive coil inputs into an AD8421 low-noise instrumentation amplifier with a gain of 3. An LTC6263 differential Op-Amp forms a low-pass filter, configured to give a bandwidth of approximately 84 kHz.

Receive voltage and transmit current data are acquired by the ADCs within the DSP and the CLA is used to down-sample the data by a factor of 10. A fast Fourier transform is applied to the time domain data, and the components of the transmit harmonics are extracted and transmitted to a host computer via UART. A custom Python user interface provides hardware control and real-time data display. The software allows the user to view magnitude/phase or real/imaginary data as a time series, complex vector, or frequency plot as required. Data logging captures the raw packets from the DSP, allowing offline processing and analysis.

**Figure 5:**
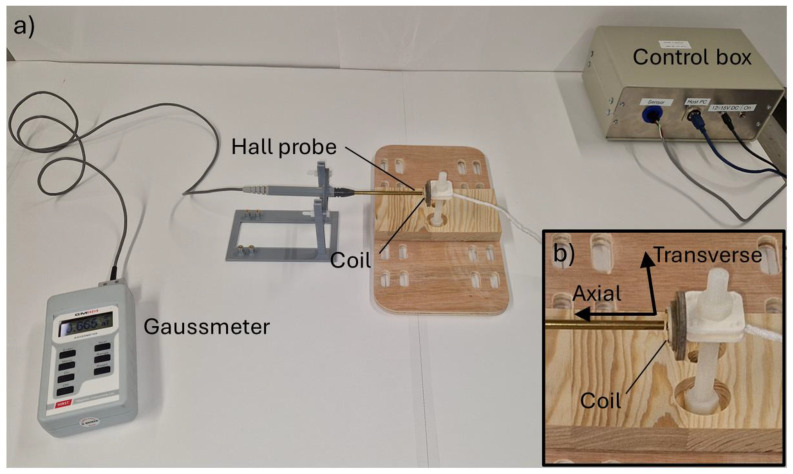
(**a**) Setup used to measure the transmit field, (**b**) close-up of Hall probe and coil showing measurement directions.

The primary magnetic field generated by the transmit coils was measured using a Hirst GM08 Gaussmeter with axial and transverse hall probes. The probes were positioned with the probe tip approximately 0.88 mm from the sample (Figure 5), corresponding to the nominal distance between the coil and the sample’s upper surface when assembled in the DSC chamber. The field measurements were made with single- and multi-harmonic excitations, which are reported in Table 2. In order to prevent the ADC system from saturating, it was necessary to reduce the overall transmit magnitude in some of these measurements. The transmit magnitudes used in the field measurements are also given in Table 2. Due to the chamber design, it was not possible to make these measurements in situ, and so these values give only an indicative measurement of the field generated by the coil when in air. The silver bowl of the chamber will clearly have a significant effect on the generated field due to the eddy currents circulating within it. Furthermore, the GM08 gaussmeter is only rated to frequencies up to 10 kHz; therefore, the measurements of frequencies above the 10th system harmonic are only indicative.

As the DSC and electromagnetic measurement software are not currently integrated into a single package, the phase and magnitude data were synchronised with the temperature data record by the DSC during post-processing.

### 2.2. Sample Preparation

**Figure 6:**
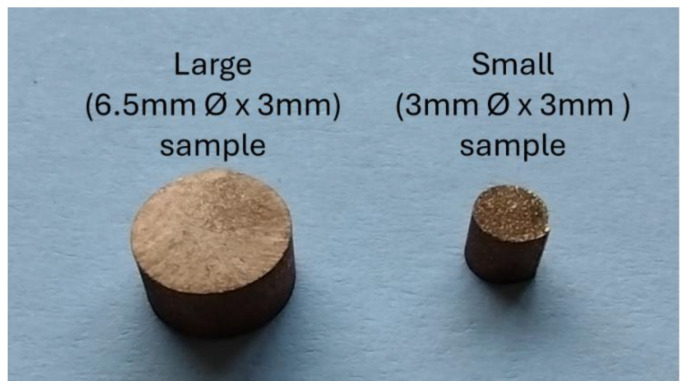
Large and small test samples.

A range of samples, like those shown in Figure 6, were fabricated for the tests in two sizes: 6.4 mm diameter × 3 mm height (large) and 3 mm diameter × 3 mm height (small). The samples were cut from rods using a cutter machine equipped with a cooling system. They were then washed with ethanol to remove any oil or grease. The large samples were designed to present the maximum possible sample area to the receive coil, thus maximising signal-to-noise ratio while still producing a plausible output from the DSC. Samples were fabricated from a range of materials, as shown in Table 3.

Although the EM response for the samples is heavily dependent on their size and shape, along with their position with respect to the transmit and receive coils, these factors remain constant for all tests. Disregarding geometrical effects, the EM response for a particular sample is dominated by two main factors: electrical conductivity and magnetic permeability. In this case, the field generated by the transmit coil is relatively low, so the low field permeability of the samples must be considered. The influence of permeability and conductivity are also frequency-dependent. At low frequencies, eddy currents generated in the samples are weak and the EM response is dominated by the magnetisation of the sample as characterised by magnetic permeability. As the frequency is increased, eddy currents in the sample become stronger and conductivity increasingly dominates the EM response.

This sample set was designed to give a range of electromagnetic responses both at room temperature and when heated. For example, copper is diamagnetic and highly electrically conductive, whereas the stainless steel is paramagnetic and has a much lower conductivity. Pure iron and mild steel have comparatively high magnetic permeability and medium conductivity, whereas nickel also has medium conductivity but lower permeability. Hence, each material should give a unique electromagnetic response at room temperature, testing the capabilities of the equipment to quantify the differing levels of electrical conductivity and magnetic permeability.

To maximise the signal-to-noise ratio for the electromagnetic signal, a larger sample size than is typically tested in the DSC is used. The large samples weigh between 600 and 800 mg (≈6.4 mm in diameter with a thickness of ≈3 mm), whereas the recommended sample weight for the DSC is 5–50 mg. To quantify the impact of the larger sample on the heat flow data and to ensure the DSC remains sensitive to phase changes, samples weighing 46 mg and 705 mg were fabricated and tested for comparison. The material chosen for the test was a bespoke steel alloy, Fe-0.5C-6Mn, designed to have a relatively low ferrite-to-austenite transformation temperature, which is within the operating temperature range of the specific DSC instrument used for these tests. JMatPro software V14.0 [27] was used to estimate the phase transformation temperature, predicting the start of the ferrite–austenite transition at around 670 °C.

**Figure 7:**
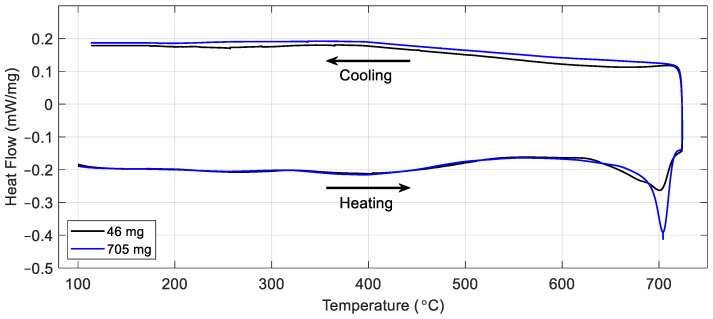
DSC data for Fe-0.5C-6Mn samples with different weights.

Figure 7 shows the results of the tests carried out on the two samples at heating and cooling rates of 5 °C per minute with a maximum temperature of 720 °C. It can be seen from the plot that once the heat flow was calibrated to the sample weight, the signals for the two samples followed a similar path, with a signal feature corresponding to the phase transition peaking at around 700 °C. The −0.41 mW/mg maximum heat flow measured for the large sample size equates to an absolute value of around −290 mW, well within the ±500 mW dynamic measurement range quoted for this instrument [25]. It can be seen from the results of this test that the large sample size can be used to study weak transitions like the ferrite-to-austenite transformation shown here, or the heat-induced changes expected in the other materials shown in Table 3, while remaining within the dynamic range of the DSC. Therefore, to maximise the signal from the sample and increase the likelihood that EM property changes could be detected by the sensor, the larger sample size was chosen for these tests. Assessment of EM techniques with a smaller sample size, more suited to the DSC, will be undertaken in future work.

## 3. Results

### 3.1. Room Temperature Tests

**Figure 8:**
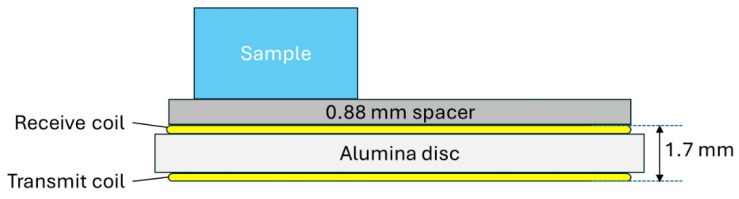
Test setup for room-temperature laboratory tests.

To assess the performance of the EM sensor coil in an ideal environment, without the presence of the metal components of the DSC, a room-temperature test was set up as shown in Figure 8. The EM sensor coil was positioned on a non-conductive surface with a 0.88 mm thick spacer used to provide the same lift-off of the sample to the sensor when installed in the DSC. As with the DSC sensor, the receive coil was positioned closest to the sample, with the transmit coil on the other side of the alumina disc. Data were acquired at three excitation frequencies simultaneously (29 kHz, 49 kHz, and 71 kHz) with and without the sample in place. The measurement acquired without the sample in place (air measurement) was subtracted from the sample measurement to give the change in magnitude (ΔMag) and change in phase (ΔPh) for each sample at each frequency. By subtracting an air measurement acquired immediately before each sample measurement, potential noise sources such as drift in the electronics or environmental factors can be negated, allowing for a more accurate assessment of the capabilities of the measurement coil in this idealised case.

**Figure 9:**
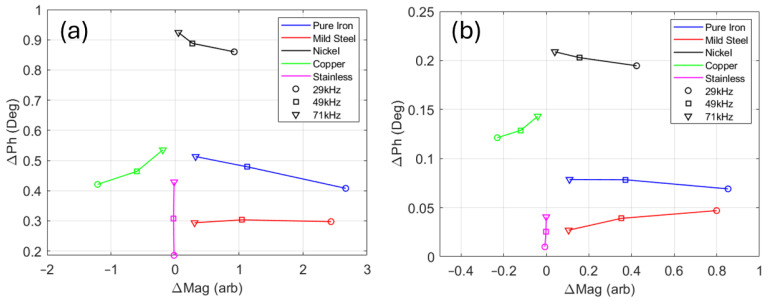
Room-temperature testing. Change in magnitude vs. change in phase for large samples (**a**) and small samples (**b**).

Figure 9 shows the results of the tests. It can be seen from the plot for the large samples (Figure 9a) that placing the ferromagnetic, high-permeability, pure iron, and mild steel samples 0.88 mm above the receive coil resulted in a large increase in magnitude and a small increase in phase. The lower-permeability nickel invoked a smaller increase in magnitude and a much larger increase in phase. In contrast, introducing the diamagnetic, high-conductivity copper sample caused a decrease in magnitude and a similar phase shift to the mild steel and iron. For the paramagnetic, low-conductivity stainless steel sample, the positive phase shift was reduced in comparison to the copper, and the change in magnitude was very small.

Figure 9a also shows a clear trend in ΔMag with frequency. The lowest-frequency (29 kHz) data points showed the greatest change in magnitude for all samples, except for the stainless steel. The greatest change in magnitude for variation in frequency was exhibited by the pure iron and mild steel. These two materials had much higher magnetic permeability than the rest of the samples; hence, there was a decrease in ΔMag as the frequency increased and the influence of permeability on the magnitude of the signal decreased. Examination of the change in phase with frequency shows that, for the paramagnetic and diamagnetic samples (stainless steel and copper), ΔPh increased as frequency increased, with the higher-conductivity copper sample having a larger overall ΔPh, indicating some correlation between ΔPh and electrical conductivity. However, this correlation does not hold true for the ferromagnetic samples.

The plots for the small samples (Figure 9b) showed similar distributions for the five materials, but with much reduced amplitude in both ΔMag and ΔPh. This test showed that the electromagnetic measurement system developed for the DSC installation can discriminate well between a range of samples with different electromagnetic characteristics and should be able to detect the relatively small thermally induced changes in permeability and conductivity in the DSC.

**Figure 10:**
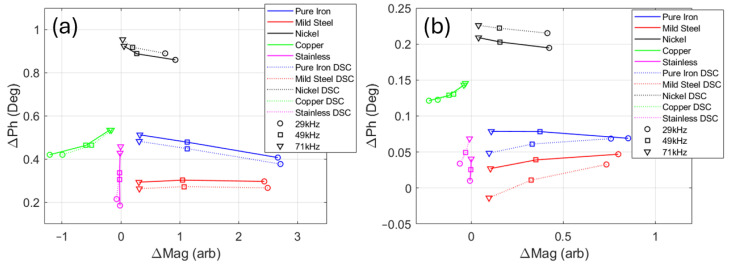
Room-temperature testing in DSC. Change in magnitude vs. change in phase for large samples (**a**) and small samples (**b**), where the solid lines are for tests outside the DSC and dashed lines are for tests inside the DSC.

One further test was conducted to assess the influence of the metal components in the DSC (see Figure 1c) on the electromagnetic measurements. The same set of samples was tested at room temperature at the same frequencies, but with the samples in situ in the DSC as they would be for a high-temperature test. The results of the test are shown in Figure 10, along with the results from the room-temperature test outside the DSC for comparison. It can be seen from Figure 10a that, for the large samples, the measurements made with the samples inside the DSC (dashed lines) followed the same trend as the measurements made outside the DSC (solid lines). The results for the small samples in the DSC (Figure 10b) also followed the same trends, but with reduced ΔMag and ΔPh and slightly more scatter, as might be expected when the receive coil is presented with a much smaller volume of target material.

It should be noted that identical results were not expected for the tests inside and outside the DSC, as the large volume of highly conductive silver in the DSC bowl was expected to have some effect on the sensor output, but it does seem that the signal from the silver did not saturate the sample signal, allowing different sample behaviours to be distinguished.

### 3.2. High Temperature Test on Nickel Sample

DSC testing was conducted at heating and cooling rates of 10 °C per minute to a maximum temperature of approximately 600 °C, with a nitrogen gas purge with a flow rate of 50 mL/minute. EM signals were recorded at the same time as the DSC data, with the DSC temperature and time interpolated and matched with the EM time to allow the EM data to be plotted with respect to the temperature recorded by the DSC. From all the samples shown in Table 3, pure nickel was selected for high-temperature testing due to its low Curie temperature (TC) between 353 and 360 °C [28], which is within the operational range of the DSC and should result in a strong change in the EM signal.

**Figure 11:**
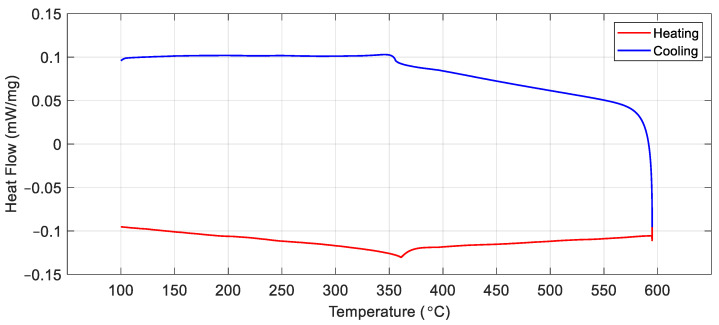
DSC data for a pure nickel sample with a heating and cooling rate of 10 °C/min.

Figure 11 shows the DSC measurement for the nickel sample. Although the heat capacity change associated with the nickel Curie transition is very small [29], there was a clear inflection in the heat flow plot at 360 °C during heating and 355 °C during cooling, corresponding to the points at which nickel transitioned from a ferromagnetic to a paramagnetic state upon heating and back again upon cooling. The 5 °C thermal hysteresis at the ferromagnetic transition of the sample was not intrinsic, but due to thermal lags inherent to DSC measurements.

**Figure 12:**
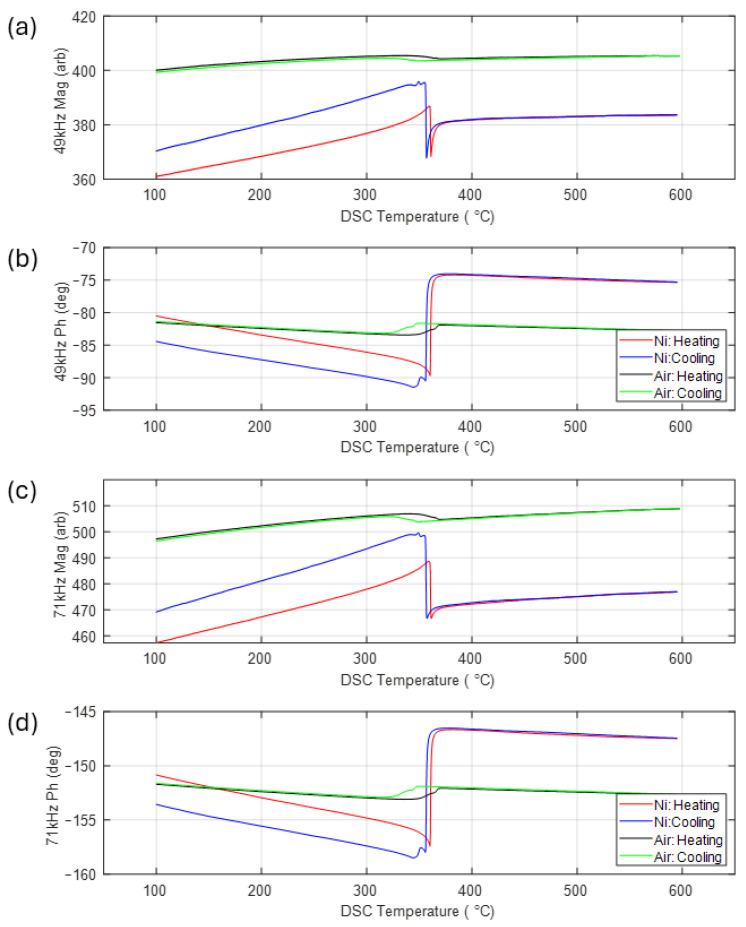
EM signal data plotted with respect to the temperature recoded by the DSC for a nickel and no (air) sample. (**a**) Magnitude at 49 kHz, (**b**) phase at 49 kHz, (**c**) magnitude at 71 kHz, (**d**) phase at 71 kHz.

Figure 12 shows the EM signal data from the ceramic coils installed in the DSC. EM measurements were recorded at two frequencies (49 kHz and 71 kHz) simultaneously, with the recorded data expressed in terms of the magnitude of the receive signal and the difference in phase between the transmit and receive signals for the two frequencies. In contrast to the room-temperature tests shown in Figure 9 and Figure 10, where air measurements were subtracted from the sample data, absolute magnitude and phase are shown here.

As well as the data from the test with the nickel sample, data from a separate test with an empty DSC chamber (identified as ‘Air’ in the plot) are shown for comparison. It can be seen from Figure 12 that the air measurement was not completely flat, as might be expected; rather, there was a small increase in the magnitude and a decrease in phase as the temperature increased and a reversal of this trend upon cooling. This was most likely caused by the interaction between the signal from the coil and the silver bowl (see Figure 1c), which formed part of the furnace of the DSC. As the bowl heated up during the test, the conductivity of the silver decreased, causing a change in the signal recorded by the receive coil. Superimposed on the slight incline in the plots for the air measurement, there was also a signal feature at around 350 °C. In addition to the silver DSC bowl and the titanium screws holding the ceramic coils in place, there were two other metal components in the near vicinity of the coil which could be the source of this signal feature. The constantan sample stand was directly below the coil, in the bottom of the DSC bowl (see Figure 1c), and the thermocouple wires were directly above the coil, embedded in the inner ceramic cap (see Figure 2). Both were exposed to heating and both had a significant nickel content; therefore, the feature at approximately 350 °C could have been due to the Curie temperature effect for these Ni-rich metallic components. There was also a small amount of nickel in the ceramic coil ENEPIG surface finish, which may have also contributed. As this background signal was relatively small, no attempt was made to compensate for its influence on the signal from the samples.

Examination of the plots for the nickel sample in Figure 12 shows a large change in both magnitude and phase at approximately 360 °C upon heating and 355 °C upon cooling, reflecting the 5 °C thermal hysteresis also seen in the DSC results for this sample (Figure 11). As with the DSC results, these changes in the EM signal correspond to the transformation of nickel from a ferromagnetic to a paramagnetic material and back again. Although there was a large shift in the overall signal level between the two frequencies, the trends in the data were very similar, with the Curie transition for increasing temperature corresponding to a sharp decrease in signal magnitude and a similarly sharp increase in phase. These abrupt signal features were superimposed on a steady increase in magnitude (decrease in phase) up to the TC and a much smaller increase in magnitude (decrease in phase) above TC.

The interaction between the EM field and the sample can be explained in terms of three main factors. The first is a steady increase in magnetic permeability approaching TC on heating, followed by a large drop in permeability when TC is reached [30]. This change in permeability is the primary source of the increase in magnitude (decrease in phase) approaching TC and the large change in the EM signals when TC is reached. The second factor is a monotonic increase in the electrical conductivity of the nickel as temperature increases [30]. This is the most likely source of the slight incline in magnitude (decline in phase) above TC. The third factor is a large increase in electromagnetic skin depth caused by the large decrease in permeability at TC and the corresponding change in the distribution of the EM field in the sample.

Although the change in the EM response to the heating of the nickel sample above TC is reversible (it follows the same path on heating and cooling), the same cannot be said for the EM response below TC, with the magnitude returning to a much higher value and the phase to a much lower value upon cooling back to 100 °C. Although the nickel sample returned to a ferromagnetic state upon cooling, this does not mean that the microstructure was the same before and after the test. To assess the changes in the nickel sample, microstructural features in the sample were examined using EBSD scans. An Oxford EBSD system, connected to a JEOL JSM-7800F0 scanning electron microscope with an accelerating voltage of 20 kV, was employed for this purpose. The step sizes of the EBSD scans ranged from 0.075 to 0.5 μm, depending on the size of the features being analysed. The EBSD samples were prepared through mechanical polishing, employing 0.05 μm colloidal silica for the final polishing stage. The microstructure was determined using AZtecCrystal software V3.1.

**Figure 13:**
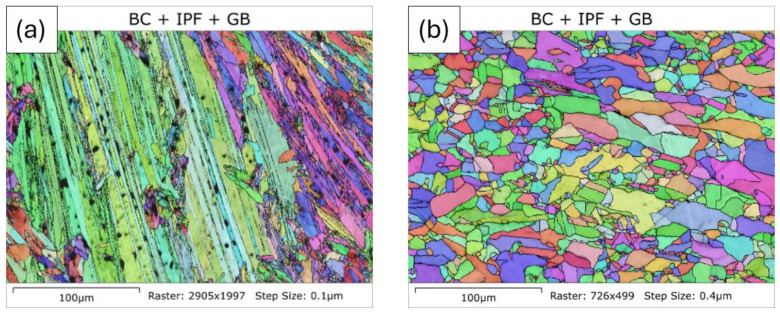
Microstructure of the as-received pure nickel sample before (**a**) and after (**b**) the DSC experiment. Sample heated to 600 °C and cooled to 100 °C, with a heating and cooling rate of 10 °C per minute.

Figure 13 shows an EBSD image of the microstructure of the nickel sample before and after heating. It can be seen that heating the nickel quite significantly changed the microstructure, with modification to the grain size and dislocation density in the material. Both of these factors had an effect on the EM response to the ferromagnetic properties of nickel [28], explaining why the nickel sample looked electromagnetically different after cooling to 100 °C.

## 4. Discussion and Conclusions

This paper details the development of an EM measurement system for installation in a DSC. The EM measurement system consists of a specially designed coil printed onto a ceramic substrate and suspended under a ceramic cap in the DSC. The coil is interfaced with bespoke data acquisition hardware and used to carry out multifrequency measurements of EM magnitude and phase while a sample is heated in the DSC and simultaneous heat flow measurements are taken.

The performance of the ceramic coil was first evaluated at room temperature outside the DSC to eliminate interference from metallic DSC components, and then measurements were taken with the EM sensor coil in the DSC. Room-temperature tests demonstrated that the coil can successfully discriminate between samples with different electromagnetic properties both outside and inside the DSC, showing clear correlations between variations in magnetic permeability and electrical conductivity and variations in EM magnitude and phase.

Finally, the combined DSC-EM device was used to perform simultaneous DSC heat flow and multifrequency EM measurements on a pure nickel sample heated to 600 °C. Test results show that the EM signal data provided a clear, reliable and repeatable indicator of the Curie temperature and the associated magnetic phase transition of nickel, validating the capability of the combined DSC-EM instrument to provide complementary data to characterise changes in material microstructural properties.

The work shows that the combined DSC-EM instrument can significantly enhance DSC characterisation capabilities, providing a more comprehensive understanding of metallurgical changes. The EM sensor coil approach has the additional advantage in that, because it is non-contact, it can be incorporated into standard DSC equipment without significant changes to the apparatus and with negligible impact to the DSC measurements themselves.

Future work will involve further refinement of the EM measurement system, eliminating the contamination of the sample response by signals from the silver DSC bowl and other metallic components, either by subtraction of the EM signal obtained by heating an empty DSC bowl or by employing a differential measurement coil. Work will be carried out to exploit the multi-frequency capabilities of the apparatus to convert the measured signals into meaningful parameters such as magnetic permeability and electrical conductivity. Further tests will be carried out on a variety of materials, testing the capabilities of the DSC-EM instrument to assess different microstructural changes.

## Figures and Tables

**Table 1:** Transmit waveform configuration.

Component Harmonic	Relative Magnitude	Relative Phase
29	0.5	0.0
49	0.5	0.0

**Table 2:** Magnetic field measured at 0.88 mm above the coil surface.

Transmit Harmonic	RMS Magnetic Field [mT]		Relative Transmit Magnitude
	Axial	Transverse	
1	1.51	0.47	0.3
5	1.21	0.47	0.3
10	1.61	0.51	0.5
29	1.11	0.54	0.85
41	0.76	0.50	0.85
29 + 41 (50%—50%)	0.66	0.48	0.85

**Table 3:** Sample dimensions and properties.

Material	Large Sample Size (mm)	Small Sample Size (mm)	Chemical Composition	Comments
Pure Iron	6.41Ø × 2.96	2.94Ø × 2.98	Fe ~99.8%	High permeability, medium conductivity
Mild Steel	6.36Ø × 2.90	2.95Ø × 2.84	Fe-0.15C-0.6Mn	High permeability, medium conductivity
Nickel	6.41Ø × 2.90	3.07Ø × 2.90	Ni ~99%	Medium permeability, medium conductivity
Copper	6.25Ø × 3.01	3.05Ø × 2.77	Cu ~99%	Diamagnetic, high conductivity
Stainless Steel (316)	6.40Ø × 2.94	2.99Ø × 2.93	Fe-16Cr-10Ni-2Mo	Paramagnetic, low conductivity
Med Steel (Fe-0.5C-6Mn)	6.40Ø × 2.95	2.98Ø × 2.92	Fe-0.5C-6Mn	Medium permeability, low to medium conductivity

## Data Availability

Data supporting the conclusions of this article will be made available by the authors upon request.
